# Biological and Environmental Influences on Parturition Date and Birth Mass of a Seasonal Breeder

**DOI:** 10.1371/journal.pone.0124431

**Published:** 2015-04-17

**Authors:** Daniel M. Wolcott, Ryan L. Reitz, Floyd W. Weckerly

**Affiliations:** 1 Texas State University, Department of Biology, San Marcos, Texas, United States of America; 2 Texas Parks and Wildlife Department, Kerr Wildlife Management Area, Hunt, Texas, United States of America; University of KwaZulu-Natal, SOUTH AFRICA

## Abstract

Natal features (e.g. Julian birth date and birth mass) often have fitness consequences and can be influenced by endogenous responses by the mother to seasonal fluctuations in nutritional quality and photoperiodic cues. We sought to further understand the biological and environmental factors that influence the natal features of a polytocous species in an environment with constant nutritional resources and limited seasonal variation. During a 36-year study we assessed the influence of biological factors (maternal age and litter type [i.e., litter size and sexual composition]) and environmental factors (total precipitation and mean maximum temperature during months encompassing conception, the last trimester of gestation, and the entire length of gestation) on Julian birth date and birth mass using linear-mixed effects models. Linear and quadratic functions of maternal age influenced both natal features with earliest Julian birth dates and heaviest birth masses occurring at prime-age and older individuals, which ranged from 5–9 years of age. Litter type influenced Julian birth date and birth mass. Interestingly, environmental factors affected Julian birth date and birth mass even though mothers were continuously allowed access to a high-quality diet. Random effects revealed considerable variation among mothers and years. This study demonstrates that, in long-lived polytocous species, environmental factors may have a greater influence on natal features than previously supposed and the influence from biological factors is also complex. The documented responses to environmental influences provide unique insights into how mammalian seasonal reproductive dynamics may respond to current changes in climate.

## Introduction

The amount of body development that an individual achieves in the first year of life is fundamental to survival in long-lived species [1–4]. The amount of body development that is possible during that period is a function of mass at birth and quality of nutritional resources available to the mother [5–7]. For many populations of mammals, seasonality in food supplies leads to birth synchrony, which is often necessary to time parturition to annual pulses of high-quality nutritional resources [8–10]. If an individual is born too early, body development necessary for survival may not be complete and survival probabilities during the first few weeks after parturition become greatly reduced [11,12]. If an individual is born too late in spring, growth rates and low-quality nutrition limit the ability for that individual to reach a body mass necessary for overwinter survival [13,14]. Thus, timing of parturition to conditions that are most amenable for survival and growth of young is often a function of conception date or adjustment of gestation length [15–17].

Much of the current theory about seasonal breeding is contingent on maternal condition and photoperiod [6,18–20]. Maternal condition is controlled by a number of factors including social rank [21], age [22], previous breeding experience [23], individual variation [24–26], and nutrition [27–29]. Litter type (i.e., litter size and sexual composition) also has been shown to affect length of gestation and timing of parturition. In sexually dimorphic, monotocous species, gestation length is often longer, parturition dates later, and birth mass heavier for males [1]. In polytocous species, however, complications from both sex and size of litter on parturition date have led to inconclusive findings [30–31]. Much of maternal condition is dependent on the ability of an individual to obtain nutritional resources as efficiently as possible, with prime-age individuals often having the most success [32]. Because of this, litter size and birth mass is often influenced by maternal age in a quadratic manner, with prime-age individuals producing the largest litter sizes and heaviest offspring [18,19,33].

Timing of reproduction in mammals has evolved as a response to seasonal availability of resources at high latitudes, where there is a strong correlation between photoperiod and plant growth. Because of this correlation, it is possible to use photoperiod as a predictive cue to time late gestation and parturition for when nutritional resources are most available. The mammalian neuro-endocrine pathways use photoperiodic and metabolic information from the individual to stimulate the reproductive processes in preparation for breeding [34]. However, as the latitude decreases toward the equator, environmental stochasticity increases and photoperiod is less correlated with environmental conditions conducive for reproduction [34]. Thus, Bronson [34] has suggested that endogenous responses to photoperiod should not be as strong in long-lived mammalian species at latitudes < 30° and that other environmental factors must be used in low latitudes as cues in order to maintain seasonal breeding in populations.

While a substantial amount of research has focused on understanding factors that affect parturition and survival of neonates in regard to environmental settings and maternal condition [5–7], most of these studies are confounded by variation in available nutritional resources and strong endogenous responses to photoperiodic cues [3,20]. Current findings suggest that long-lived populations of mammals at high latitudes are becoming increasingly affected by changes in global climate through trophic mismatch [35]. Thus, it is important to understand how these populations can adjust to changes in seasonality through cues other than photoperiodism as environmental conditions at high latitudes become increasingly similar to conditions at low latitudes. Thus, holding nutritional resources constant in an environment where photoperiod is less influential would be useful to more fully understand the biological and environmental factors that influence natal features of a long-lived species. By providing a constant high-quality diet, factors that may generally be swamped by high variation in nutrition and strong endogenous responses to photoperiodic cues may be more accurately assessed.

Our study provides a unique opportunity to understand the factors that influence natal features of long-lived species. For 36 years, a known-age population of captive white-tailed deer was fed a high-quality diet at a latitude with benign winter conditions. Within this population of captive white-tailed deer, measurements of parturition date (Julian birth date) and birth mass were recorded for all live births. Further, the study site was located, latitudinally, at the transitional zone suggested by Bronson [34] in which environmental stochasticity reduces correlations between nutrition and photoperiod. Given the conditions of nutritional quality in this study, we predicted that environmental cues would not be influential to these well-conditioned mothers and that only biological factors would be used by mothers to adjust Julian birth date and birth mass. Our study had two main objectives. First, we sought to understand the biological influences of litter type and maternal age on Julian birth date and birth mass. Because maternal experience is often a large component of reproduction, we postulated that maternal age would influence both Julian birth date and birth mass, whereas litter type only would be influential in affecting birth mass. The second objective was to assess the role of both biological and environmental influences (precipitation and temperature during the period of conception, the period encompassing the last trimester of gestation, and the entire length of gestation) on Julian birth date and birth mass. By controlling for biological factors known to influence natal features and maternal nutrition, we predicted that Julian birth date and birth mass would not be affected by environmental conditions. Further, any environmental conditions found to significantly influence Julian birth date or birth mass could yield important information on how high-latitude species may adapt seasonal reproductive cues in the presence of changes in high-latitude climates.

## Materials and Methods

### Ethics Statement

Prior to the initiation of the study, all animal research was reviewed and approved by the Institutional Animal Care and Use Committee (IACUC) of the United States Department of Agriculture (USDA) Animal and Plant Health Inspection Service (APHIS). Statutes of the Animal Welfare Act were followed by Kerr Wildlife Management Area personnel and registered with the USDA APHIS Animal Care permit# 74-R-0146.

### Study Area

The study was conducted at the Donnie E. Harmel White-tailed Deer Research Facility (hereafter pens) located on the Kerr Wildlife Management Area in Kerr County, Texas, USA (30° 5.2’ N, 99° 30.4’ W; S1 Table). During the study, the facility was comprised of five to seven mating pens and three to eight rearing pens. The pens were enclosed by 2.7-m high-game fencing with an area of 0.3 ha for each mating pen and 0.5–1.6 ha for each rearing pen. Vegetation in the pens was limited to non-palatable herbaceous plants such as common horehound (*Marrubium vulgare*) and cowpen daisy (*Verbesina encelioides*), as well as scattered live oak (*Quercus virginiana*) trees, which had been browsed out of reach of white-tailed deer since 1974 [36]. Nutrition for all deer ≥1.5 years of age was comprised of ad libitum access to water and food pellets (16% minimum crude protein and 18.5% acid detergent fiber) as well as a source of roughage (peanut hay or alfalfa hay) provided weekly (<1 kg per animal per week, [37]). The nutritional quality of the diet was sufficiently high to account for both body maintenance and lactation costs for all individuals, because there was no difference in post-lactation body condition between individuals that reared zero, one, or two young [37]. Each pen contained one or more feeding troughs and one watering trough.

From 1974–2012, several types of breeding programs occurred at the pens; however, much of the study program was the same throughout this time span. At ≥1.5 years of age, female deer were separated into one of five to seven mating pens. Each October, one male (average age = 3.5, range = 1.5–10.5) was placed into each mating pen and allowed to mate with all available females until the end of December, when the male was removed. Within one day of parturition, neonates were captured, individually marked, and weighed to the nearest 0.1 kg. After weaning, fawns were placed into rearing pens where they spent the next year of development, until the female portion was reintroduced into the mating pens at 1.5 years of age. Deer were initially collected from several ecological regions in Texas, establishing the original stock in 1974. Five sires collected from the Edwards Plateau ecological region were added to the closed research herd in 2007. Because all individuals were uniquely marked, ages were known for all females. To reduce the number of categorical variables describing the heterogeneity of breeding programs, all programs were fitted into three main program types. In the first program (study program 1), no animals experienced any nutritive stress and were fed a continuous 16% crude protein diet throughout their lifetime. The second program (study program 2) was a selective breeding program in which sires were selected as breeders if their first set of antlers possessed spike antler characteristics. The last program (study program 3) consisted of sires that encountered nutritive stress (8% crude protein) from 0.5–1.5 years of age and were given the ad libitum high-quality diet thereafter.

**Fig 1:**
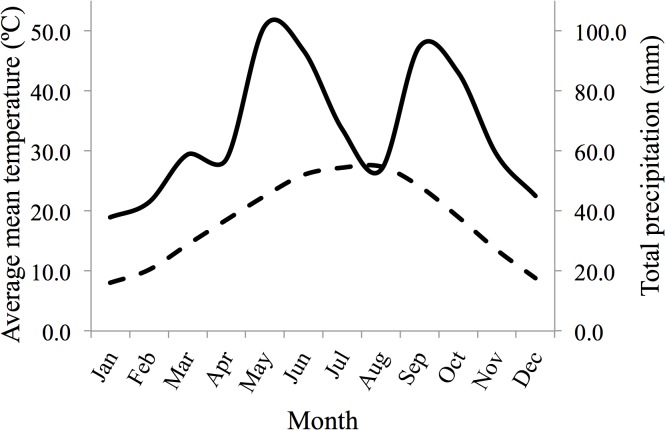
Walter climate diagram derived from a weather station in Kerrville, TX, USA from 1977–2012. The solid line represents the average total precipitation (mm) and the dashed line represents the average mean temperature for each month.

Environmental factors were collated from a weather station in Kerrville, TX, USA (30° 4.0’ N, 99° 7.0’ W), which was located approximately 38 km from the pens. Precipitation variables consisted of monthly total precipitation during periods deemed important to conception and parturition of neonates (October–December and April–June). We included two additional predictor variables by summing the monthly total precipitation from October–June (prior to conception and throughout gestation) and April–June (encompassing the months surrounding the last trimester of gestation). Months encompassing the last trimester of gestation were deemed important because most fetal growth occurs during this period [38]. Predictor variables for mean maximum temperature were calculated similarly to those of total precipitation. Mean maximum temperature was recorded for the individual months of October–December and April–June, and was also averaged between the months of October–June and April–June. Mean maximum temperature was used because it can affect activity patterns and rumination time in ungulates [39,40]. Average annual precipitation at the pens, during the study, was 802.9 mm (min = 333.1 mm, max = 1298.7 mm, *SE* = 38.5 mm, *CV* = 0.30) with most precipitation occurring from May–June (average = 195.0 mm) and September–October (average = 180.8 mm, Fig 1). Winters were mild, with mean minimum temperatures in January of 0.6°C, and summers were hot with mean maximum temperatures in August of 34.2°C.

### Statistical Analyses

During this study, females had the opportunity to breed several times throughout their lifetime. Because of this, mixed-effects models were used in all analyses with unique identifiers for both maternal identity and birth year included as random effects. Since <5% of births during this study consisted of triplets, only births of singletons and twins were included in analyses. Also, since there were only two births by females 14 years or older, these births were removed from all analyses. A Kolmogorov-Smirnov test was first conducted to test for normality in Julian birth dates [41]. Linear mixed-effects models were used for both response variables (Julian birth date and birth mass) to initially determine which biological factors significantly influenced each response variable. Subsequently, model selection analyses were utilized which included the biological factors from the previous analyses and also incorporated different possible timeframes of environmental influences on the two response variables.

Biological factors included in the analyses for both response variables were maternal age, the quadratic term for maternal age, and litter type as well as the nuisance variable for study program. The quadratic term for maternal age was included to assess the possibility of a reproductive threshold or senescence in older deer [42–44]. To determine which form of the quadratic term was present, a subset of the data containing all individuals at the apex of the quadratic term and older were used to determine whether reproductive senescence was present for each response variable [45]. If neither the linear nor quadratic term for age was significant in elderly individuals, reproductive senescence was absent and a reproductive threshold was present. This post-hoc analysis was included in all analyses in which the 95% CI for the quadratic term on age did not overlap 0. Litter type was partitioned differently depending on the response variable. In the Julian birth date analysis, one individual from each twin litter was removed to avoid doubling a Julian birth date. Litter types in the Julian birth date analysis were categorized as F = singleton female, M = singleton male, FF = twin females, MM = twin males, and FM = twin mixed litter. For the birth mass analysis, all individuals were included in the analysis and, thus, litter type was further categorized as F1 = singleton female, M1 = singleton male, F2 = females from twin female litters, M2 = males from twin male litters, FMix = females from mix litters, and MMix = males from mix litters.

We then evaluated linear mixed-effects models for both response variables that assessed the added influence of environmental factors. In each model, we included the biological variables with statistically significant *F-ratios* (*P* < 0.05) in the previous analysis (maternal age, litter type, and study program) with environmental variables (total precipitation or average maximum temperature) that were present during the period when dams could conceive, and encompassing the last trimester of gestation. A total of 19 regressions were built that assessed environmental influences from October–December (influence on conception), April–June (influence encompassing the last trimester of gestation), and October–June (influence throughout gestation). The first regression included only the biological factors that significantly influenced the two response variables in the previous analyses. All further models considered these biological influences with additional environmental influences added as covariates. The next set of six regressions assessed the influence of precipitation on the two response variables by incorporating individual months into each regression (i.e., October, November, December, April, May, and June). We then considered three more regressions by summing total precipitation during the possible length of conception (October–December), the period encompassing the last trimester of gestation (April–June), and the entire length of gestation (October–June). The next set of nine regressions followed the same design as noted previously, but included mean maximum temperatures instead of total precipitation as a predictor variable.

All statistical analyses were conducted in R version 3.0.2 [46]. We analyzed linear mixed-effects models with the lme4 package [47]. For both the Julian birth date and birth mass analyses, the model that best explained variation in Julian birth date or birth mass was selected with the Akaike Information Criterion (AIC_*c*_, [48]). Model averaging was conducted in the AICcmodavg package [49], and was used when competing models were <2 ΔAIC_*c*_ units different. Maximum likelihood estimation was used to calculate parameters during the model-selection process. Parameter estimates and 95% CI for the selected model were then reported with restricted maximum likelihood estimation [50]. Coefficients of determination were calculated for each linear mixed-effects model by calculating the variance explained by the fixed factors (marginal *R* ^*2*^) and by fixed and random factors (conditional *R* ^*2*^, [51]). In analyses where model averaging was conducted, marginal and conditional *R* ^*2*^ values were calculated using the standard deviations for the fixed and random effects derived from the model with the smallest AIC_*c*_.

## Results

**Fig 2:**
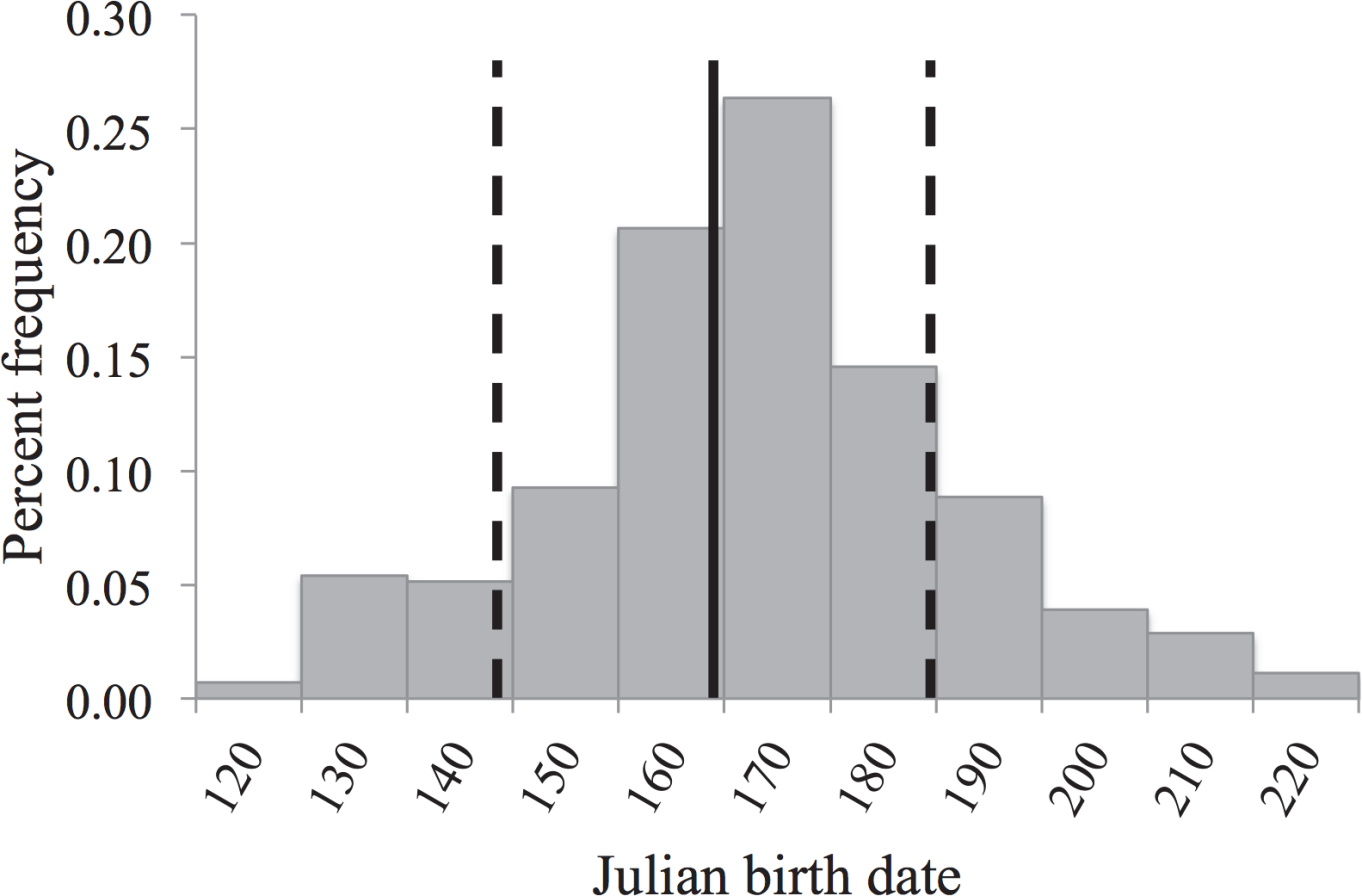
Percent frequency of Julian birth dates for litters (*n* = 1,403) of captive white-tailed deer at Kerr Wildlife Management Area, Kerr County, Texas, USA from 1977–2012. The vertical, solid line represents the mean Julian birth date (164, 13 June) and the vertical, dashed lines represent the standard deviation (144 and 185, 24 May and 4 July, respectively).

During the 36-year study of white-tailed deer, 2,290 neonates were born to 510 individual mothers for a total of 520 singletons (222 females and 298 males) and 885 twin litters (193 female twin litters, 243 male twin litters, and 449 mixed twin litters). Number of births per individual female varied during the study, with an average of 4.5 young born during the lifetime of an individual mother (min = 1, max = 17, *SE* = 0.2). Average maternal age was 4 years (*SE* = 0.05), with a range of 2–13 years. The mean date of parturition during our study was 13 June (Julian date = 164, *SE* = 0.5), with the earliest birth occurring on 13 April (Julian date = 103) and the latest on 10 September (Julian date = 253). A Kolmogorov-Smirnov 1-sample test revealed that dates of Julian birth were normally distributed during this study (*D* = 0.527, *P* = 0.944, Fig 2). Average neonate body mass was 2.6 kg (min = 0.7, max = 6.1, and *SE* = 0.01).

**Fig 3:**
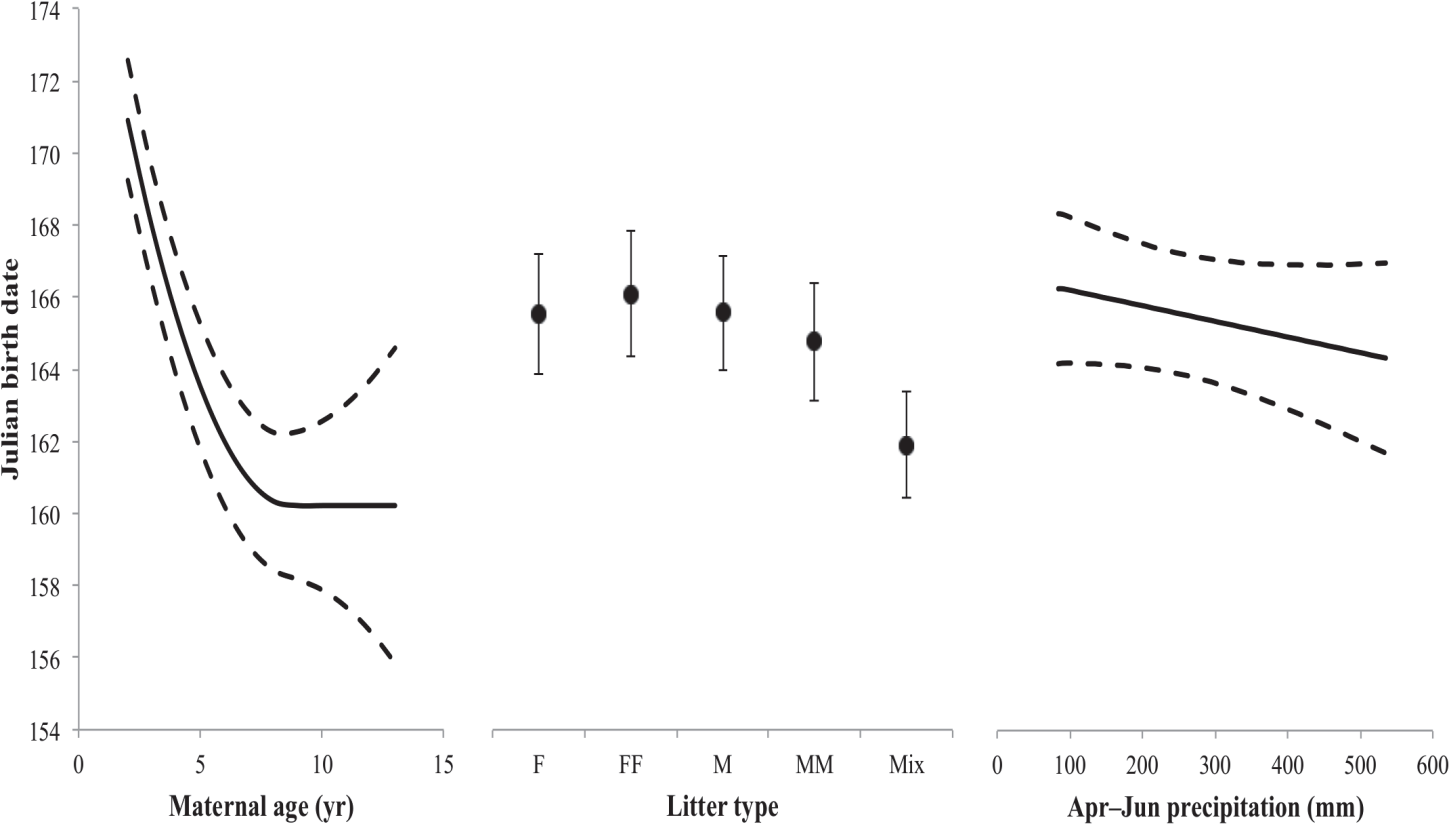
Predicted values from a linear mixed effect model estimating the parturition date (in Julian days) of captive white-tailed deer at Kerr Wildlife Management Area, Kerr County, Texas, USA from 1977–2012. Regression coefficients were obtained from model-averaged parameter estimates of competing models. Predicted Julian birth date was estimated across the range of each variable deemed important while controlling for all other variables (variable constants included: Maternal age = 4, Litter type = female singleton, Study program = study program 1, April–June precipitation = 256.2 mm, October–Jun precipitation = 611.7 mm, June precipitation = 102.6 mm, and November temperature = 20.4°C). The solid lines represent the predicted estimate for Julian birth date and the dashed lines are the standard error envelopes for the estimates. Random effects were treated as categorical variables and included a unique identifier for each mother and the year of birth for each fawn.

All biological factors tested (maternal age and litter type) influenced Julian birth date, as well as the nuisance variable (study program, Table 1). Inclusion of environmental factors, in the model-selection analysis, indicated that several models fit the data equally well. Five models were within two ΔAIC_*c*_ of each other (Table 2). Model averaged estimates of the five models indicated that maternal age had a negative relationship on Julian birth date, with every 1 year increase in maternal age decreasing Julian birth date by 4.1 days (CI = –5.7 to—2.5, Table 3). The quadratic term for maternal age increased Julian birth date as maternal age increased, with the earliest predicted birth dates occurring at 9 years of age (Fig 3). The post-hoc analysis assessing the possibility of a threshold or senescent effect after prime age demonstrated that linear and quadratic terms were not significant (*F*_1,59_ = 0.434, *P* = 0.513 and *F*_1,59_ = 0.398, *P* = 0.531, respectively), thus, there was a threshold effect. On average, mothers in the youngest age class (2 years of age) gave birth on the latest dates (171, 20 June) and the oldest mothers (13 years of age) gave birth to individuals around the same time as a 6-year old mother (160, 9 June). Predicted values of Julian birth date for each litter type indicate that mixed-sex litters were born earlier than all other litter types (Julian birth date = 161.9, *SE* = 1.5). Julian birth dates for all other litter types (F = 165.5, *SE* = 1.7; FF = 166.1, *SE* = 1.7; M = 165.6, *SE* = 1.6; and MM = 164.8, *SE* = 1.6) were not significantly different from each other (Fig 3). While the summary from the model-averaged regression included parameter estimates of environmental influences for November temperature, June precipitation, and October–June precipitation, the only 95% CI that did not overlap 0 was April–June precipitation. Julian birth date was influenced by April–June precipitation with every 1 mm increase in precipitation decreasing Julian birth date by 0.02 days. April–June precipitation was highly variable during the study (min = 85.2 mm, max = 543.6 mm, average = 253.9 mm, *CV* = 0.51). Variance components for the random effects in the Julian birth date analysis could not be derived from the model-averaged analysis, thus, we reported values derived from the model with the lowest AIC_*c*_ (M10, biological factors and April–June precipitation as main effects). Variance components for this model were dam id (*SD* = 11.26), birth year (*SD* = 4.85), and residual error (*SD* = 15.22). The marginal *R* ^*2*^ for this model was 0.09 and the conditional *R* ^*2*^ was 0.45. Inclusion of the random effects (dam id and birth year) and fixed effects explained more variation in birth mass than fixed effects alone.

**Table 1:** Sources of variation utilizing restricted maximum likelihood estimation for a linear mixed-effects model assessing the influence of reproductive components on parturition date (Julian date) of singleton and twin white-tailed deer at Kerr Wildlife Management Area, Kerr County, TX from 1977–2012.

SOV	MS	df_N_	df_D_	*F*	*P*
LitType	1093.8	4	1235	3.397	0.009
MaternalAge	9892.9	1	1241	24.560	<0.001
MaternalAge^2^	2093.3	1	1243	10.487	0.001
StudyProgram	3150.5	2	571	13.692	<0.001

Headers denote the source of variation (SOV), mean squares (MS), degrees of freedom for the numerator and denominator (df_N_, df_D_), F-test (*F*), and p-value (*P*). Sources of variation included LitType (litter types comprised of singleton female and male, twin females and males, and twin mixed litters), MaternalAge (known maternal age) and its quadratic term, and StudyProgram (grouped study programs consisted of StudyProgram1 = 16% protein diet throughout life, StudyProgram2 = sires possessed spike antler characteristics when they were 1.5 years of age, and StudyProgram3 = sires consumed 8% protein diet from 0.5–1.5 years of age and then placed on 16% protein diet for the rest of life). Random effects consisted of dam identification and year of birth.

**Table 2:** Models analyzed and summaries of model selection for the influence of biological variables (litter type, maternal age, study program), and environmental variables on parturition date (Julian date) of penned white-tailed deer from the Kerr Wildlife Management Area, Kerr County, TX from 1977–2012.

Model	Predictors	K	AIC_*c*_	ΔAIC_*c*_	weight
M10	() + Apr–JunPrecip	13	12110.1	0.0	0.17
M12	() + NovTemp	13	12110.4	0.3	0.14
M7	() + JunPrecip	13	12110.6	0.5	0.13
M9	() + Oct–JunPrecip	13	12111.8	1.7	0.07
M1	(LitType + MaternalAge + MaternalAge^2^ + StudyProgram)	12	12112.0	1.9	0.07
M5	() + AprPrecip	13	12112.4	2.3	0.05
M4	() + DecPrecip	13	12113.3	3.2	0.03
M17	() + Oct–DecTemp	13	12113.4	3.3	0.03
M6	() + MayPrecip	13	12113.4	3.3	0.03
M16	() + JunTemp	13	12113.6	3.5	0.03
M2	() + OctPrecip	13	12113.7	3.6	0.03
M13	() + DecTemp	13	12113.8	3.7	0.03
M3	() + NovPrecip	13	12113.8	3.7	0.03
M14	() + AprTemp	13	12113.8	3.7	0.03
M15	() + MayTemp	13	12113.9	3.8	0.03
M19	() + Apr–JunTemp	13	12114.0	3.9	0.03
M8	() + Oct–DecPrecip	13	12113.9	3.8	0.03
M11	() + OctTemp	13	12114.0	3.9	0.02
M18	() + Oct–JunTemp	13	12114.0	3.9	0.02

Each model contained predictor variables for litter type (LitType), age of the mother and its quadratic term (MaternalAge), study program (StudyProgram) and environmental predictors for each model. Precipitation and temperature values for each month as well as summed (precipitation) and average (temperature) total from Aug–Jun and Apr–Jun. Precipitation was calculated as the total precipitation in a month (mm). Temperature was calculated as the mean maximum temperature per month (°C). The number of parameters in each model is K, AIC_*c*_ is the Akaike value for each model, ΔAIC_*c*_ is the change in value compared to the most highly selected model and Weight is the Akaike weight for each model. Models are arranged from highest to lowest Akaike weight.

**Table 3:** Parameter estimates utilizing restricted maximum likelihood estimation for a linear mixed-effects model assessing the influence of biological (litter type, maternal age, and study program) and environmental factors on parturition date (Julian date) of singleton and twin white-tailed deer at Kerr Wildlife Management Area, Kerr County, TX from 1977–2012.

Coefficient	Estimate	SE	Lower 95% CI	Upper 95% CI
(Intercept)	185.032	9.194	167.007	203.058
LitTypeFF	0.569	1.678	–2.722	3.860
LitTypeM	0.038	1.518	–2.940	3.016
LitTypeMix	–3.622	1.409	–6.386	–0.857
LitTypeMM	–0.757	1.604	–3.903	2.390
MaternalAge	–4.103	0.830	–5.731	–2.475
MaternalAge^2^	0.234	0.072	0.092	0.376
StudyProgram2	7.814	2.087	3.720	11.907
StudyProgram3	–4.319	1.644	–7.544	–1.094
Apr–JunPrecip	–0.015	0.007	–0.029	–0.0005
NovTemp	–0.937	0.481	–1.880	0.006
JunPrecip	–0.022	0.012	–0.045	0.0008
Oct–JunPrecip	–0.007	0.004	–0.015	0.002

Litter types (LitType) were comprised of singleton female and male (F, M), twin females and males (FF, MM), and Mix (twin mixed sex). MaternalAge and its quadratic term was the known age of the mother. StudyProgram consisted of grouped study programs (StudyProgram1 = 16% protein diet throughout life, StudyProgram2 = sires possessed spike antler characteristics when they were 1.5 years of age, and StudyProgram3 = sires consumed 8% protein diet from 0.5–1.5 years of age and then placed on 16% protein diet for the rest of life). Precipitation (mm) was calculated for each month or range of months by summing total precipitation over the defined time span. Temperature (°C) was calculated as the average maximum temperature for the defined month. Random effects consisted of dam identification (*SD* = 11.26), year of birth (*SD* = 4.85), and residual variance (*SD* = 15.22).

**Fig 4:**
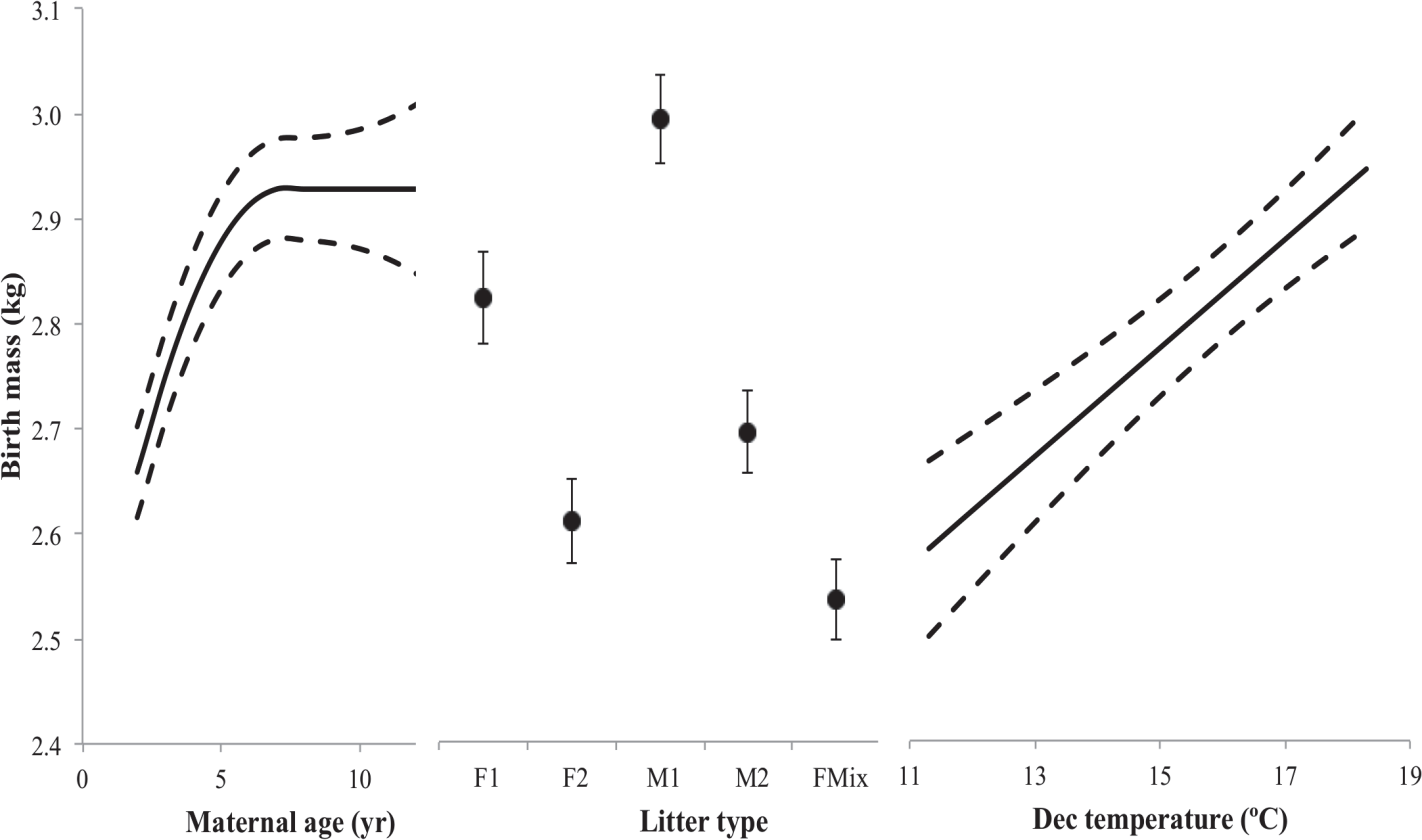
Predicted values from a linear mixed effect model estimating the birth mass (kg) of captive white-tailed deer at Kerr Wildlife Management Area, Kerr County, Texas, USA from 1977–2012. Predicted birth mass was estimated across the range of each variable deemed important while controlling for all other variables (variable constants included: Maternal age = 4, Litter type = female singleton, Study program = study program 1, and December temperature = 16.0°C). The solid lines represent the predicted estimate for birth mass and the dashed lines are the standard error envelopes for the estimates. Random effects were treated as categorical variables and included a unique identifier for each mother and the year of birth for each fawn.

Results for the birth mass analysis demonstrated that all biological factors influenced birth mass (Table 4). Inclusion of environmental factors, in the model selection analysis, suggested that December temperature explained the most variation in birth mass (Table 5). A summary of the selected model (Table 6) demonstrated that maternal age and precipitation each had a positive relationship on birth mass, with every 1 unit increase in maternal age and temperature (year, °C) increasing birth mass by 0.14 and 0.05 kg, respectively (Fig 4). The quadratic term for maternal age decreased birth mass as maternal age increased with the heaviest predicted birth masses (2.9 kg) occurring at 5 years of age (Fig 4). The post-hoc analysis assessing the possibility of a threshold or senescent effect after prime age revealed that linear and quadratic terms were not significant (*F*_1,756_ = 0.058, *P* = 0.810 and *F*_1,756_ = 0.058, *P* = 0.810, respectively), thus, there was a threshold effect. The lightest birth masses occurred at 2 years of age and heaviest at 5 years of age and older. Predicted values of birth mass for each litter type demonstrated that birth mass varied significantly among litter types. Females from mixed litter types had the lowest birth mass (FMix, 2.54 kg, *SE* = 0.04) followed by females of twin litters (F2, 2.61 kg, *SE* = 0.04), males of mixed litters (MMix, 2.68 kg, *SE* = 0.04), males of twin litters (M2, 2.70 kg, *SE* = 0.04), females of singleton litters (F1, 2.82 kg, *SE* = 0.04), and the heaviest birth masses were males of singleton litters (M1, 2.99 kg, *SE* = 0.04). Total birth mass for singleton and twin litters varied with total birth mass of singleton litters weighing less than twin litters (F1 = 2.82 kg, M1 = 2.99 kg, F2 = 5.22 kg, Mix = 5.22 kg, and M2 = 5.40 kg). Variance components for the random effects in the birth mass analysis were dam id (*SD* = 0.28), birth year (*SD* = 0.14), and residual error (*SD* = 0.41). The marginal *R* ^*2*^ was 0.12 and the conditional *R* ^*2*^ was 0.44. Inclusion of the random effects (dam id and birth year) along with the fixed effects explained more variation in birth mass than fixed effects alone.

**Table 4:** Sources of variation utilizing restricted maximum likelihood estimation for a linear mixed-effects model assessing the influence of reproductive components on birth mass (kg) of singleton and twin white-tailed deer at Kerr Wildlife Management Area, Kerr County, TX from 1977–2012.

SOV	MS	df_N_	df_D_	*F*	*P*
LitType	6.2	5	2160	40.994	<0.001
MaternalAge	8.2	1	2258	59.280	<0.001
MaternalAge^2^	5.7	1	2256	36.442	<0.001
StudyProgram	1.0	2	965	5.933	0.003

Headers denote the source of variation (SOV), mean squares (MS), degrees of freedom for the numerator and denominator (df_N_, df_D_), F-test (*F*), and p-value (*P*). Sources of variation included LitType (litter types comprised of singleton female and male, twin females and males, and twin mixed litters), MaternalAge and its quadratic term (known age of the mother), and StudyProgram (grouped study programs consisted of StudyProgram1 = 16% protein diet throughout life, StudyProgram2 = sires possessed spike antler characteristics when they were 1.5 years of age, and StudyProgram3 = sires consumed 8% protein diet from 0.5–1.5 years of age and then placed on 16% protein diet for the rest of life). Random effects consisted of dam identification and year of birth.

**Table 5:** Models analyzed and summaries of model selection for the influence of biological variables (litter type, maternal age, study program), and environmental variables on birth mass (kg) of captive white-tailed deer from the Kerr Wildlife Management Area, Kerr County, TX from 1977–2012.

Model	Predictors	K	AIC_*c*_	ΔAIC_*c*_	weight
M13	() + DecTemp	13	3026.8	0.0	0.82
M2	() + OctPrecip	13	3032.6	5.8	0.05
M6	() + MayPrecip	13	3033.9	7.1	0.02
M17	() + Oct–DecTemp	13	3034.7	7.9	0.02
M11	() + OctTemp	13	3034.8	8.0	0.02
M1	(LitType + MaternalAge + MaternalAge^2^ + StudyProgram)	12	3035.3	8.5	0.01
M5	() + AprPrecip	13	3036.1	9.3	0.01
M18	() + Oct–JunTemp	13	3036.6	9.8	0.01
M8	() + Oct–DecPrecip	13	3036.7	9.9	0.01
M12	() + NovTemp	13	3037.0	10.2	0.01
M14	() + AprTemp	13	3037.2	10.4	0.01
M16	() + JunTemp	13	3037.2	10.4	0.01
M4	() + DecPrecip	13	3037.1	10.3	0.01
M7	() + JunPrecip	13	3036.9	10.1	0.01
M9	() + Oct–JunPrecip	13	3037.3	10.5	0.00
M10	() + Apr–JunPrecip	13	3037.3	10.5	0.00
M15	() + MayTemp	13	3037.3	10.5	0.00
M19	() + Apr–JunTemp	13	3037.3	10.5	0.00
M3	() + NovPrecip	13	3037.3	10.5	0.00

Each model contained predictor variables for litter type, age of the mother, and study program and added predictors for each model are shown below. Precipitation was calculated as the total precipitation (mm) in a month or range of months. Temperature was calculated as the mean maximum temperature (°C) per month or range of months. Number of parameters in each model is K, AIC_*c*_ is the Akaike value for each model, ΔAIC_*c*_ is the change in value compared to the most highly selected model and Weight is the Akaike weight for each model. Models are arranged from highest to lowest Akaike weight.

**Table 6:** Parameter estimates utilizing restricted maximum likelihood estimation for a linear mixed-effects model assessing the influence of biological (litter type, maternal age, and study program) and environmental factors on birth mass of white-tailed deer at Kerr Wildlife Management Area, Kerr County, TX from 1977–2012.

Coefficient	Estimate	SE	df	Lower 95% CI	Upper 95% CI
(Intercept)	1.591	0.250	29	1.102	2.077
LitTypeF2	–0.213	0.039	2196	–0.290	–0.137
LitTypeFMix	–0.288	0.037	2154	–0.361	–0.216
LitTypeM1	0.170	0.040	2181	0.092	0.249
LitTypeM2	–0.128	0.038	2210	–0.202	–0.054
LitTypeMMix	–0.142	0.037	2154	–0.215	–0.070
MaternalAge	0.140	0.018	2237	0.105	0.176
MaternalAge^2^	–0.010	0.002	2238	–0.013	–0.007
StudyProgram2	–0.102	0.049	790	–0.196	–0.007
StudyProgram3	–0.118	0.038	622	–0.191	–0.041
DecTemp	0.052	0.015	27	0.022	0.082

Litter types (LitType) were comprised of singleton female and male (F, M), twin females and males (FF, MM), and Mix (twin mixed sex). MaternalAge was the known age of the mother and MaternalAge^2^ was its quadratic term. StudyProgram consisted of grouped study programs (StudyProgram1 = 16% protein diet throughout life, StudyProgram2 = sires possessed spike antler characteristics when they were 1.5 years of age, and StudyProgram3 = sires consumed 8% protein diet from 0.5–1.5 years of age and then placed on 16% protein diet for the rest of life). DecTemp was calculated as the average maximum temperature (°C) in the month of December. Random effects consisted of dam identification (SD = 0.28), year of birth (SD = 0.14), and residual variance (SD = 0.41).

## Discussion

This study assessed the influences of biological and environmental factors on natal features (Julian birth date and birth mass) of a long-lived, polytocous species. The first objective was to understand the biological influences of litter type and maternal age on Julian birth date and birth mass. As expected, the biological factors of maternal age and litter type affected both of these natal features. The second objective was to assess the influence of environmental factors on Julian birth date and birth mass. Interestingly, we found that environmental conditions influenced these natal features even while holding nutritional resources constant and accounting for maternal traits. Total precipitation during the months that encompassed the last trimester of gestation (April–June) influenced Julian birth date, and December temperatures influenced birth mass.

Natal features are commonly dependent on biological factors related to age, social rank, nutritional condition, and previous breeding experience of mothers. We found that maternal age influenced both natal features until 5–9 years of age, which is considered prime to late-prime age for this species [52]. There was no evidence of senescence after prime age was exceeded, which was most likely a function of the high quality and easily digested forage that was available ad libitum. In polytocous species, we expected Julian birth date would be later for litter sizes larger than one offspring to accommodate for the reduced allocation of resources to a particular offspring. Our findings suggest that mothers with a mixed litter type tended to give birth earlier with no statistical differences among any other litter type. When considering the influence of litter type on birth mass, results from this study are similar to other studies on free-ranging polytocous species, with males and females from singleton litters weighing more than individuals of either sex from twin litters [31,53,54]. Recent studies have suggested that prenatal hormonal interactions between fetuses in twin litters influence birth mass [53,55,56]. Disparity in birth mass between females and males in mixed and same-sex twin litters (2.5 kg and 2.7 kg, respectively) in our study further confirms that maternal and fetal influences have a role in allocating resources amongst twin fetuses [57]. Hormonal interactions that affect birth mass may also be an underlying cause for the unexpected earlier birth dates of mixed litters, but more work is needed to fully understand causation.

Based on the differences in study program during the 36 years, study program was considered a confounding variable that could influence natal features. We had no *a priori* expectation for study program and found an influence among study programs on Julian birth date and birth mass. Factors within study programs that could influence natal features might include genetic characteristics and age of the sire (study program 2) or reduction of nutritional quality to sires at 0.5–1.5 years of age (study program 3). Because of study-design constraints in the current study, it was not feasible to utilize sire traits as informative variables. The inclusion of sire as a random effect could have been beneficial to explain some of the variation in natal features. However, each sire was, generally, only bred during a single year and, thus, it was more appropriate to allow the variation among sires to be accounted for within the study program nuisance variable.

In many environments, precipitation and temperature are drivers of primary productivity [58] that directly affect the nutritional quality of forage for herbivores. While environmental conditions have been shown to positively influence neonatal post-parturition survival in ungulates, evidence of environmental influences on parturition date or birth mass has been limited to old world cervids (Cervinae, [59]). Lack of evidence for environmental influences on seasonal reproduction in new world ungulates has been largely attributed to endogenous responses to photoperiod as a predictor for seasonal dynamics in vegetation. Bronson [34] suggested that environmental influences should be more influential at latitudes <30° because of reduced correlations between photoperiod and seasonal dynamics in vegetation. Indeed, at a similar latitude, reduced seasonality and benign winter conditions were recently shown to influence body development among cohorts in a long-lived species differently than in studies at higher latitudes [60].

Because of the constant high level of nutritional quality in this study, we anticipated that environmental influences would not be important predictors of Julian birth date or birth mass. However, mothers still used precipitation during the period encompassing the last trimester of gestation as a cue for favorable environmental conditions in which to birth their young, and birth mass was influenced by temperature during the period closer to conception. Interestingly, these two natal features are influenced at different periods of gestation rather than being connected to each other (i.e., parturition occurring only after favorable birth mass is achieved). These findings suggest that the presumably well-conditioned mothers were exhibiting risk-sensitive reproductive allocation in both Julian birth date and birth mass [61–63]. Mothers used precipitation cues during the latter stages of gestation to determine favorable environmental conditions for rearing young. Neonates are more vulnerable than adults to inclement weather, and climatic settings indirectly influence mothers through availability of nutritional resources to provision young during the energetically demanding time of lactation [10,64]. Further, mothers can use temperature during the winter months to determine how to best allocate resources for overwinter survival and birth mass [65]. Since all mothers were presumed to be well-conditioned, there should be no concern over food availability throughout their lifetime. However, these findings suggest an endogenous or innate response to environmental cues was still present. This innate response from well-conditioned mothers may also explain differences in variation explained by the fixed and random effects in our analyses.

In a large-scale study on reproductive seasonality in captive ruminants in zoological parks, Zerbe et al. [20] reported no evidence for environmental influences on parturition date when controlling for nutrition. This outcome may be due to resolution issues caused by their calculations of breeding peaks (greatest number of births within 5 days of each other), because small interannual variation in precipitation or temperature may be swamped out by a breeding peak analysis. In our analysis, April–June precipitation decreased the Julian birth date by 0.02 days for every 1 mm increase in precipitation. While this regression coefficient seems small, the high interannual stochasticity at our study area led to a 7-day adjustment in Julian birth date across the range of possible precipitation values. Given a daily natal growth rate of 0.24 kg [66], early born individuals could increase their body mass by 1.7 kg over the 7-day period and be approximately 50–70% heavier than late-born individuals.

Although environmental and biological factors were influential predictors of Julian birth date and birth mass, only 9% of the variation in Julian birth date and 12% of the variation in birth mass was explained by those factors. With the inclusion of the random effects for dam id and birth year, the explanation of variation increased to 45% and 44%, respectively. This result indicates that it is necessary to account for subject-specific effects, because of individual variation in longitudinal studies. Recently, much emphasis has been placed on the role of individual variation in maternal care [19,25]. Those studies suggested that some mothers were naturally better at producing healthy fawns, even in poor environmental conditions. This appears to be true in the current study as well. Further, it is often assumed that supplemental feeding of ungulates will reduce interanimal variability by increasing the overall condition of the herd [67]. Our study demonstrates that with a high nutritional plane, individual variation of the mothers still greatly influences these natal features and actually accounts for more of the variation than any biological or environmental influence. The random effect for birth year was also necessary in explaining variation in Julian birth date and birth mass. This random effect aided in accounting for interannual variation because of unidentified latent variables that affect maternal condition (e.g., density of deer in pens or disease).

Understanding factors that influence natal features in long-lived species is often difficult because nutritional state of mothers is affected by recruitment of young from previous reproductive events and climatic heterogeneity [17,28,63]. By controlling for nutrition, in an environment with limited photoperiodic influence, our study demonstrates that both biological and environmental factors influenced natal features of a long-lived polytocous species. These natal features, in turn, affect future survival and reproductive performance. How these factors influence natal features is quite complicated and is dependent on maternal and paternal experience as well as environmental influences at both conception and late-term gestation. This study highlights the ability of a long-lived seasonal breeder to use environmental conditions as a cue for reproductive timing and development. As high-latitude climates become increasingly similar to low-latitude climates, this information is necessary to more fully understand how long-lived mammals may adapt to changing conditions.

## Supporting Information

S1 Table Biological and environmental factors used as predictor variables in analyses on parturition date and birth mass. (XLSX)
